# Bilateral Carotid-Cavernous Fistula Following Traumatic Fall: A Case Report

**DOI:** 10.5334/jbsr.3696

**Published:** 2024-09-17

**Authors:** Fatim Camara, Chiara Mabiglia, Thomas Bonnet

**Affiliations:** 1 Radiology resident, Erasmus Hospital, Brussels, Belgium; 2 Neuroradiologist, Erasmus Hospital, Brussels, Belgium; 3 Interventional Neuroradiologist, Erasmus Hospital, Brussels, Belgium

**Keywords:** Carotid-cavernous fistulas, Traumatic brain injury, Neuro-ophthalmologic risks, Exophthalmos, Ophthalmoplegia, Magnetic resonance angiography, Digital subtraction angiography, Endovascular treatment

## Abstract

Carotid-cavernous fistulas (CCFs) are abnormal connections between the carotid arteries and the cavernous sinus, posing significant neuro-ophthalmologic risks. This report presents a rare case of bilateral post-traumatic CCFs, focusing on clinical presentation, diagnosis, and management. Symptoms mimic conjunctivitis, causing diplopia, exophthalmos, and ophthalmoplegia. Diagnosis relied on computed tomography, magnetic resonance angiography, and digital subtraction angiography. Management involved transarterial embolization with coils, achieving successful outcomes. This highlights the importance of timely intervention and comprehensive imaging to prevent complications.

*Teaching point:* This case report details a rare instance of bilateral post-traumatic carotid-cavernous fistulas, emphasizing clinical presentation, diagnostic evaluation, and management.

## Introduction

Carotid-cavernous fistulas (CCFs) are abnormal connections between the carotid artery and the cavernous sinus, causing severe neuro-ophthalmologic complications like vision loss, cranial nerve palsies, and intracranial hemorrhage. They are classified as direct, typically from trauma, or indirect, often linked to aneurysms or connective tissue disorders [1, 2]. Bilateral CCFs are rare and usually traumatic, leading to symptoms like exophthalmos, chemosis, and cranial nerve deficits [3, 4].

Prompt diagnosis and management are vital. Advanced imaging techniques, such as multidetector computed tomography (CT) and magnetic resonance (MR) angiography, are crucial for classification and treatment planning, while digital subtraction angiography (DSA) remains the gold standard [5, 6]. Modern management involves endovascular techniques like transarterial and transvenous embolization using coils, balloons, and liquid embolics, which have high success rates [7, 8].

This case report presents a rare instance of bilateral post-traumatic CCFs, emphasizing the importance of advanced imaging techniques, particularly MR imaging (MRI) and DSA, in timely identification and management.

## Case Report

**Figure 1:**
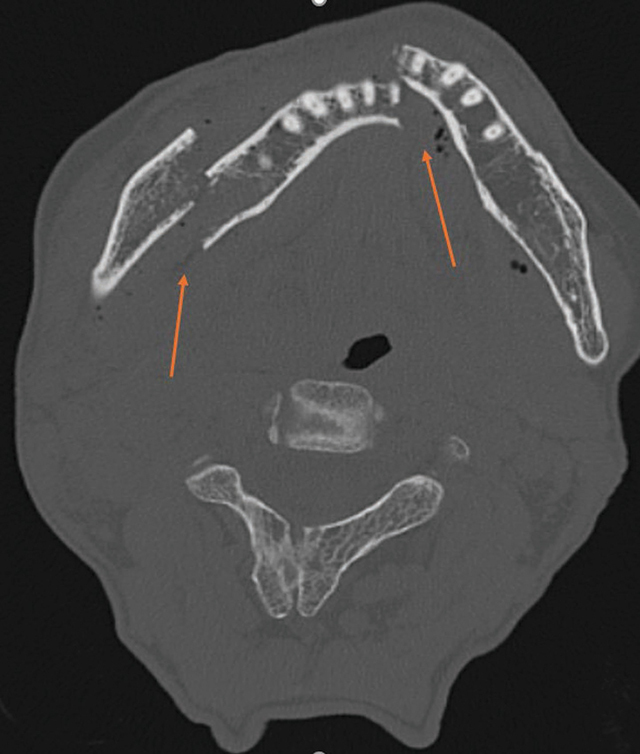
Axial CT scan showing a bifocal mandibular fracture with displacement.

**Figure 2:**
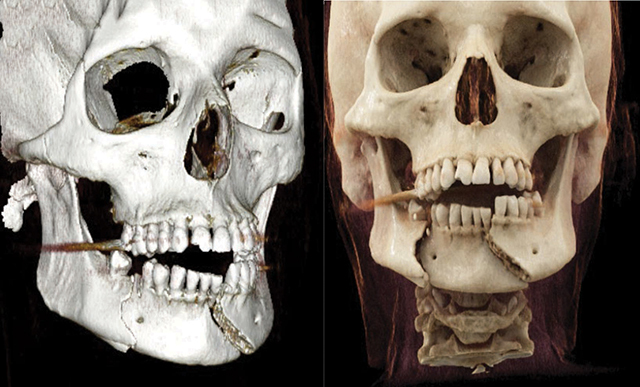
3D CT reconstruction showing a bifocal mandibular fracture with displacement.

**Figure 3:**
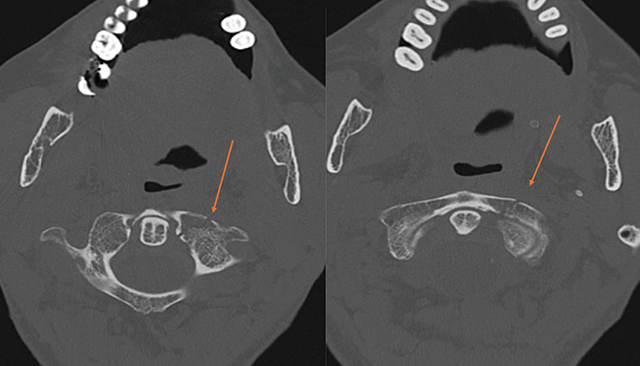
Axial CT scan (bone window) showing a non-displaced fracture of the left anterior arch of the C1 vertebra.

A 64-year-old woman fell, resulting in lip numbness and difficulty closing her mouth. CT showed a bifocal mandibular fracture and a left C1 fracture (Figs. 1–3), without intracranial bleeding, treated surgically.

Three weeks later, she developed right eye redness, swelling, and tearing, initially misdiagnosed as conjunctivitis. Bilateral diplopia and VI nerve paresis, primarily on the right, followed. CT showed grade 1 exophthalmos of the left eye without fracture.

**Figure 4:**
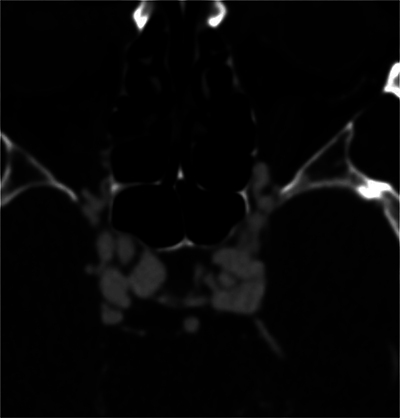
CT angiography showing early opacification of the cavernous sinuses.

**Figure 5:**
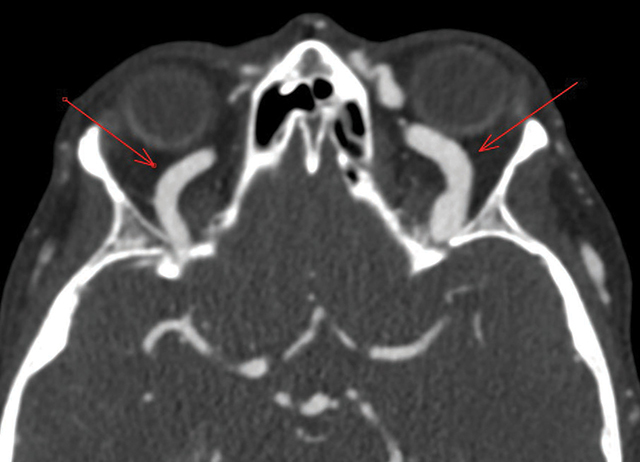
CT angiography showing dilatation of the superior ophthalmic veins.

**Figure 6:**
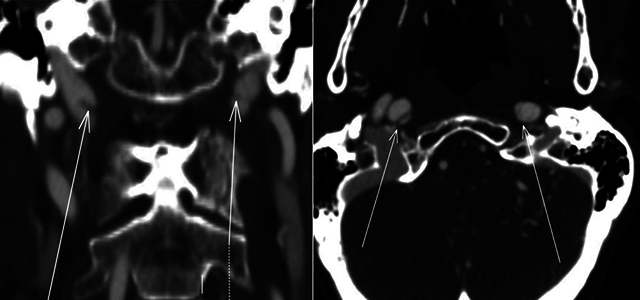
CT angiography showing sequelae of dissection in the cervical portion of the bilateral internal carotid arteries, with an intimal flap indicated by an arrow.

Post-contrast brain CT revealed enlarged intracavernous portions of the internal carotid arteries, dilated cavernous sinuses, and superior ophthalmic veins (Figs. 4 and 5), indicating a CCF and signs of dissection sequelae (Fig. 6).

**Figure 7:**
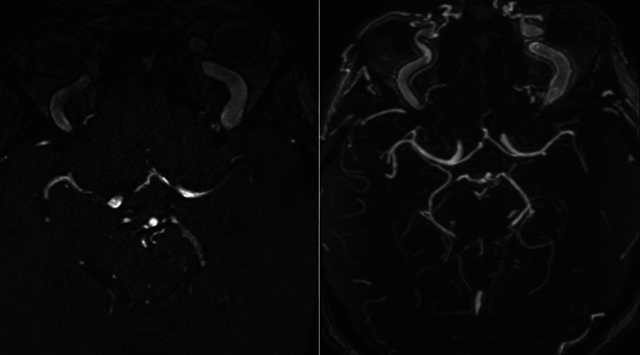
MR angiography showing dilatation of the superior ophthalmic veins.

**Figure 8:**
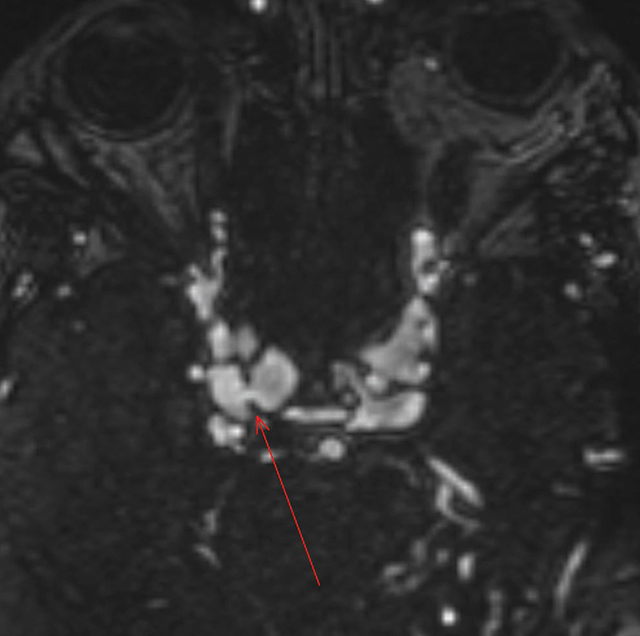
MR angiography showing a fistula between the cavernous sinus and the cavernous portion of the carotid artery.

**Figure 9:**
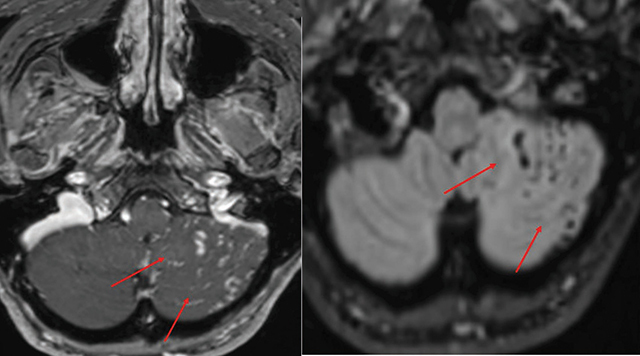
Axial T2 and post-contrast T1 weighted images showing dilatation of the leptomeningeal veins in the left cerebellar hemisphere, indicating abnormal venous drainage.

**Figure 10:**
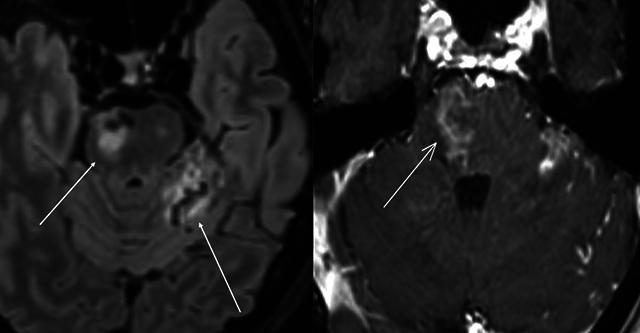
FLAIR and post-contrast T1 weighted images showing a hemorrhagic lesion in the right anterior pontine region, with surrounding edema and contrast enhancement, indicative of venous infarction.

MR angiography confirmed bilateral CCFs, showing enlarged, arterialized superior ophthalmic veins (Fig. 7), a direct arteriovenous fistula on the right (Fig. 8), and dilated leptomeningeal veins (Fig. 9). A hemorrhagic lesion in the right anterior pontine region suggested venous infarction (Fig. 10).

**Figure 11:**
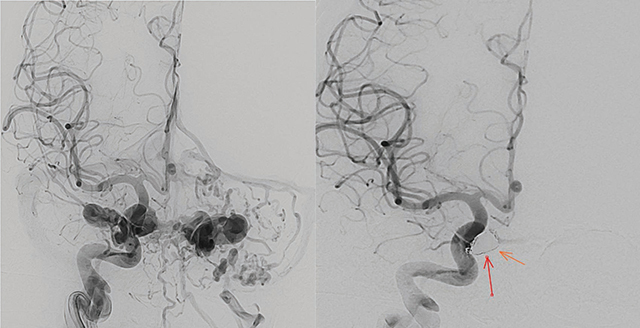
Pre-treatment Digital Subtraction Angiography (DSA) showing bilateral carotid-cavernous fistulas (CCFs), followed by post-treatment images demonstrating successful occlusion of the CCF.

DSA was used for planning treatment. Percutaneous transarterial embolization with coils was performed. Sixteen coils nearly occluded the fistula, leaving a slight shunt (Fig. 11).

**Figure 12:**
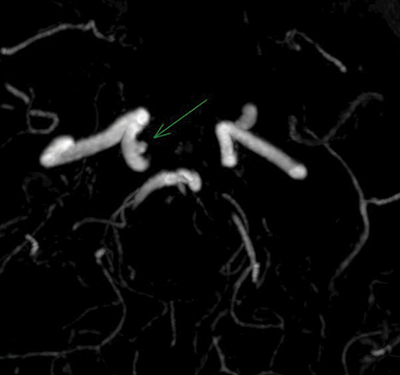
MR Angiography showing the presence of a residual fistula following treatment.

**Figure 13:**
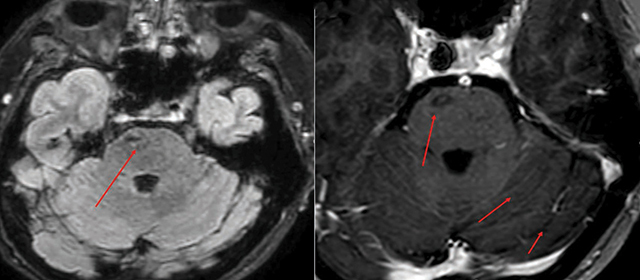
MRI images showing regression of edema in the right pontine region and persistent dilation of the cerebellar veins, indicating partial resolution of the condition.

**Figure 14:**
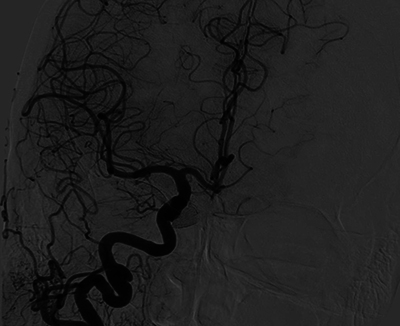
Digital Subtraction Angiography showing complete obliteration of the carotid-cavernous fistula (CCF), indicating successful treatment.

One month post-treatment, the patient reported mild diplopia. Follow-up MRI revealed a 3-mm residual shunt (Fig. 12), complete vein regression, and reduced edema around the hemorrhagic lesion (Fig. 13). DSA confirmed complete fistula obliteration (Fig. 14).

## Discussion

Bilateral CCFs are rare and complex. In this case, trauma caused tears in the internal carotid artery, leading to conjunctivitis-like symptoms with diplopia and ophthalmoplegia. These symptoms result from increased venous pressure and arterialization of conjunctival vessels, causing redness, swelling, and hyperemia [9].

The use of CT and MR angiography identified bilateral CCFs with venous dilation and arterialized flow in the superior ophthalmic veins and cavernous sinuses. MR images also showed edema, hemorrhagic changes, and venous infarctions. CCFs can cause significant vascular complications, including hemorrhages and infarctions, due to arterial blood entering the venous system, leading to venous hypertension, vessel wall weakening, and potential rupture. Additionally, venous congestion can impair drainage, causing ischemia and infarctions [2, 9].

DSA was essential for planning endovascular treatment. Percutaneous transarterial embolization, typically with detachable balloons or platinum coils, is the preferred approach. In this case, DSA guided the embolization with coils, proving invaluable for mapping the fistula and assessing the procedure’s success [2, 9].

Successful outcomes included complete CCF occlusion and regression of venous dilation and edema. However, a minimal residual shunt necessitates ongoing monitoring to prevent complication.

The complexity and rarity of bilateral CCFs demand expertise in imaging for accurate diagnosis and management. Future research should refine diagnostic and treatment strategies, with guidelines needed for long-term monitoring to prevent recurrence and complications.

This case also demonstrated that the management of CCFs involves a multidisciplinary approach, requiring collaboration among neurologists, ophthalmologists, radiologists, and interventional neuroradiologists.

## Conclusion

This case emphasizes the difficulties in diagnosing and treating bilateral CCFs, a rare and complex condition. Advanced imaging and targeted embolization were effective, leading to significant symptom improvement. However, the presence of a minimal residual shunt highlights the need for long-term follow-up. The successful outcome highlights the importance of a multidisciplinary approach for comprehensive care and improved patient outcomes.
